# 2037. Epidemiology and Preventability of Hospital Onset Bacteremia and Fungemia in two Hospitals in India

**DOI:** 10.1093/ofid/ofac492.1659

**Published:** 2022-12-15

**Authors:** Sumanth Gandra, Sanjeev Singh, Murali Chakravarthy, Merlin Moni, Pruthu Dhekane, Zubair Mohamed, Anil Kumar, Arun Kaushik, Fathima Shameen, Priyadarshini Senthil, Tejaswini Saravanan, Anu George, Dorothy Sinclair, Dustin Stwalley, Jacaranda van Rheenen, Matthew Westercamp, Rachel Mann Smith, Surbhi Leekha, David K Warren

**Affiliations:** Washington University School of Medicine in St. Louis, St. Louis, Missouri; Amrita Institute of Medical Sciences, Kochi, Kochi, Kerala, India; Fortis Hospital, Bengaluru, Bengaluru, Karnataka, India; Amrita Institute of Medical Sciences, Kochi, Kochi, Kerala, India; Fortis Hospital, Bengaluru, Bengaluru, Karnataka, India; Amrita Institute of Medical Sciences, Kochi, Kochi, Kerala, India; Amrita Institute of Medical Sciences, Kochi, Kochi, Kerala, India; Fortis Hospital, Bengaluru, Bengaluru, Karnataka, India; Amrita Institute of Medical Sciences, Kochi, Kochi, Kerala, India; Fortis Hospital, Bengaluru, Bengaluru, Karnataka, India; Fortis Hospital, Bengaluru, Bengaluru, Karnataka, India; Amrita Institute of Medical Sciences, Kochi, Kochi, Kerala, India; Washington University School of Medicine in St. Louis, St. Louis, Missouri; Washington University School of Medicine in St. Louis, St. Louis, Missouri; Washington University School of Medicine in St. Louis, St. Louis, Missouri; The Centers for Disease Control and Prevention, Atlanta, Atlanta, Georgia; CDC, Atlanta, Georgia; University of Maryland School of Medicine, Baltimore, MD; Washington University School of Medicine in St. Louis, St. Louis, Missouri

## Abstract

**Background:**

The National Healthcare Safety Network (NHSN) central line-associated bloodstream infection (CLABSI) is a widely accepted quality measure. However, studies from the United States indicate that NHSN reportable CLABSIs account for less than 20% of all hospital-onset bacteremia and fungemia (HOB, i.e., any positive blood culture obtained at least 3 calendar days after hospital admission) events and about 66% of all HOB events are potentially preventable. The incidence and overall preventability of HOB is unknown in low and middle-income countries (LMICs). This study evaluated the epidemiology and preventability of HOB in two hospitals in India.

**Methods:**

Six months data on all consecutive blood cultures processed in two hospitals (Hospital A- 8.16.2020 to 2.15.2021; Hospital B- 1.1.2021 to 6.30.2021) were collected prospectively to calculate HOB and CLABSI incidence. Correlation between HOB and CLABSI rates was assessed using Spearman’s rank correlation. Medical records of 300 consecutive HOB events were retrospectively reviewed to determine the source and preventability of HOB utilizing a structured guide developed for the study.

**Results:**

Among 3,558 hospitalized patients from whom blood cultures were obtained, 10.4% developed HOB with 409 unique HOB events (HOB incidence: 2.87 per 1000 patient-days). Only 15% (59 of 409) of HOB events were reported as CLABSI as per hospital CLABSI surveillance programs. The CLABSI rate was 4.4 per 1000 central line-days. There was a moderation correlation (r=0.51; p=0.07) between HOB and CLABSI rates. Among the 300 HOB events for which medical records were reviewed, the most common organism isolated was *Klebsiella pneumoniae* and 75% of *K. pneumoniae* were carbapenem resistant (Figure 1). The most common source of HOB was CLABSI (26.3%) (Figure 2). Fifty-two percent of all HOB events and 45% of HOB events not attributable to contaminants were potentially preventable (Figure 3). CLABSIs accounted for 69% of non-contaminant HOB preventable events.

**Conclusion:**

We found that 69% of the non-contaminant related HOB preventable events were due to CLABSI. Prevention efforts in these hospitals could focus on CLABSI to reduce HOB rates while additional studies are performed to better understand the epidemiology of HOB in LMICs.

Microorganisms identified from 300 Hospital Onset Bacteremia and Fungemia Events in Two Hospitals in India.

**Figure:**
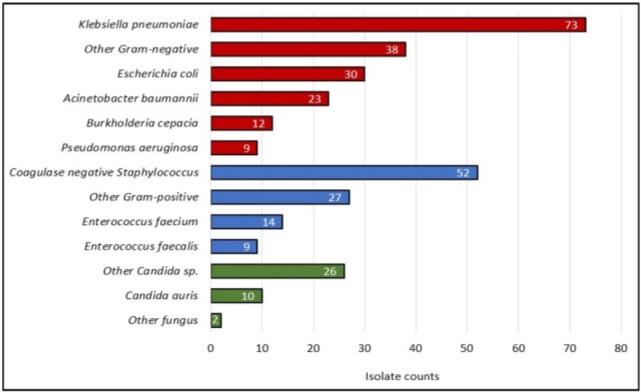

Source of Hospital Onset Bacteremia and Fungemia Events in Two Hospitals in India (n=300).

**Figure:**
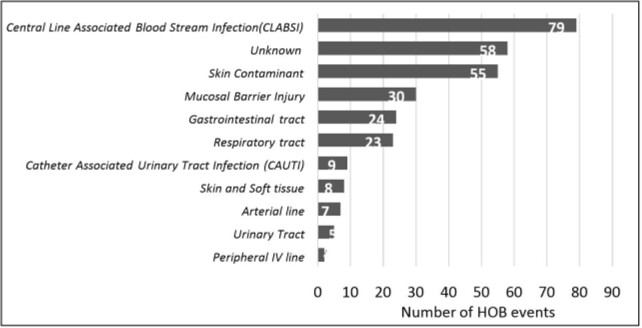

Preventability Rating Source of Hospital Onset Bacteremia and Fungemia events in Two Hospitals in India (n=300).

**Figure:**
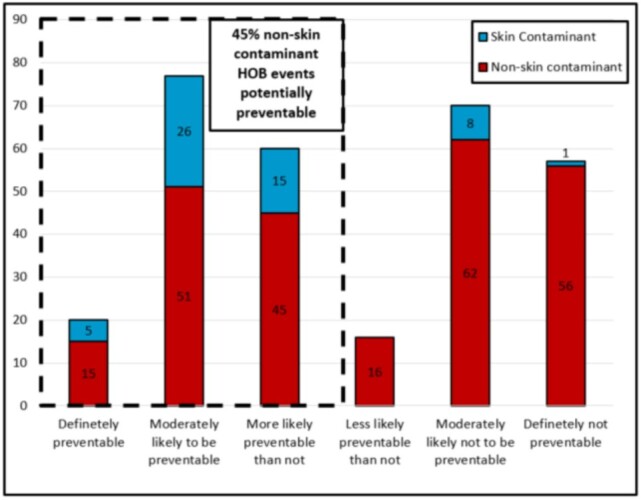

**Disclosures:**

**All Authors**: No reported disclosures.

